# State-Specific Cessation Behaviors Among Adult Cigarette Smokers — United States, 2014–2015

**DOI:** 10.5888/pcd16.180349

**Published:** 2019-03-07

**Authors:** Teresa W. Wang, Kimp Walton, Ahmed Jamal, Stephen D. Babb, Anna Schecter, Yvonne M. Prutzman, Brian A. King

**Affiliations:** 1 Office on Smoking and Health, National Center for Chronic Disease Prevention and Health Promotion, Centers for Disease Control and Prevention, Atlanta, Georgia; 2 Division of Cancer Control and Population Sciences, National Cancer Institute, Bethesda, Maryland

## Abstract

This study assessed state-specific smoking cessation behaviors among US adult cigarette smokers aged 18 years or older. Estimates came from the 2014–2015 Tobacco Use Supplement to the Current Population Survey (N = 163,920). Prevalence of interest in quitting ranged from 68.9% (Kentucky) to 85.7% (Connecticut); prevalence of making a quit attempt in the past year ranged from 42.7% (Delaware) to 62.1% (Alaska); prevalence of recently quitting smoking ranged from 3.9% (West Virginia) to 11.1% (District of Columbia); and prevalence of receiving quit advice from a medical doctor in the past year ranged from 59.4% (Nevada) to 81.7% (Wisconsin). These findings suggest that opportunities exist to encourage and help more smokers to quit.

**Summary**

**What is already known on this topic?**

Quitting smoking is one of the most important steps smokers can take to improve their health.

**What is added by this report?**

During 2014–2015, as many as 6 in 7 US adult cigarette smokers were interested in quitting smoking (state range, 68.9%–85.7%); 3 in 5 made a past-year quit attempt (42.7%–62.1%); 1 in 9 recently quit smoking (3.9%–11.1%); and 4 in 5 received advice to quit smoking in the past year from a medical doctor (59.4%–81.7%).

**What are the implications for public health practice?**

These findings underscore the importance of comprehensive state tobacco control programs and barrier-free, proactively promoted access to cessation treatments to help smokers quit successfully.

## Objective

Quitting smoking is one of the most important steps smokers can take to improve their health (1). Previous studies describing cessation behaviors among adult cigarette smokers primarily focused on national findings because state-level data are limited (2,3). To address this research gap, we used the 2014–2015 Tobacco Use Supplement to the Current Population Survey (TUS-CPS) to estimate the percentage of adult cigarette smokers in the 50 US states and District of Columbia (DC) who were interested in quitting smoking, attempted to quit within the past year, recently quit smoking, and received advice to quit from a medical doctor in the past year.

## Methods

Data came from the 2014–2015 Tobacco Use Supplement to the Current Population Survey (TUS-CPS), a cross-sectional, household-based survey of noninstitutionalized US adults aged 18 years or older in the 50 US states and DC (4). The TUS is administered with the CPS every 3 or 4 years (5). The 2014–2015 TUS-CPS was conducted in 3 waves: July 2014, January 2015, and May 2015. For all waves combined, 163,920 adults completed the interview as self-respondents (average self-response rate: 54.2%).

Current smokers were defined as adults who had smoked at least 100 cigarettes during their lifetime and currently smoked every day or some days. Former smokers were those who had smoked at least 100 cigarettes during their lifetime but currently did not smoke at all.

Current smokers indicated their interest in quitting smoking on a 10-point scale ranging from 1 (not at all interested) to 10 (extremely interested); a response from 2 to 10 was considered to indicate an interest in quitting. Current smokers who made a quit attempt in the past year reported having stopped smoking for 1 or more days or reported having made a serious attempt to stop smoking even for less than 1 day within the past year; former smokers who quit within the past year were included in this group. Recent successful smoking cessation was defined as former smokers who quit smoking within the past year and remained quit for 6 months or longer. Recent successful cessation was assessed among current smokers who initiated smoking 2 or more years ago and former smokers who quit within the past year, a definition consistent with the Healthy People 2020 definition (6). Receipt of advice to quit from a medical doctor was determined among current smokers who visited a medical doctor within the past year and among former smokers who visited a medical doctor within the year before they quit smoking.

Data were weighted to yield state-representative point estimates and 95% confidence intervals (CIs) for all 50 states and DC. Quartiles were mapped for each indicator. Statistical analyses were performed by using SAS-callable SUDAAN, version 11.0.1 (Research Triangle Institute).

## Results

During 2014–2015, the proportion of current cigarette smokers who were interested in quitting ranged from 68.9% (95% CI, 64.1%–73.6%) in Kentucky to 85.7% (81.3%–90.2%) in Connecticut (Table). For this indicator (Figure, panel A), 6 of 13 states in the lowest quartile (≤75.4%) were in the South (Kentucky, Mississippi, North Carolina, Oklahoma, Tennessee, West Virginia). In the highest quartile, 6 of 13 states (≥80.2%) were in the Northeast (Connecticut, Maine, Massachusetts, New Hampshire, New Jersey, New York).

**Table:** State-Level Prevalence of Interest in Quitting Smoking^a^, Past-Year Quit Attempts^b^, Recent Smoking Cessation^c^, and Receipt of a Medical Doctor’s Advice to Quit Smoking^d^, Tobacco Use Supplement to the Current Population Survey, United States, 2014–2015

State	Interested in Quitting^a^, % (95% CI)	Past-Year Quit Attempts^b^, % (95% CI)	Recent Smoking Cessation^c^, % (95% CI)	Receipt of Medical Doctor’s Advice to Quit^d^, % (95% CI)
Alabama	80.0 (75.8–84.2)	56.7 (52.2–61.3)	8.8 (5.6–11.9)	64.3 (59.0–69.6)
Alaska	78.5 (72.2–84.8)	62.1 (56.7–67.5)	8.2 (4.0–12.4)	70.2 (62.4–78.0)
Arizona	75.2 (70.7–79.6)	53.1 (47.7–58.4)	5.4 (3.5–7.3)	71.4 (64.3–78.5)
Arkansas	75.9 (71.8–80.0)	50.8 (46.7–55.0)	5.8 (3.4–8.2)	61.0 (55.3–66.6)
California	78.6 (75.7–81.6)	54.2 (50.9–57.5)	9.8 (7.9–11.7)	69.3 (65.4–73.3)
Colorado	79.5 (75.1–83.9)	60.7 (55.8–65.5)	10.4 (7.1–13.6)	71.6 (65.6–77.7)
Connecticut	85.7 (81.3–90.2)	59.7 (52.7–66.7)	9.0 (5.8–12.2)	74.6 (68.0–81.1)
Delaware	79.3 (73.3–85.2)	42.7 (35.9–49.5)	6.5 (3.4–9.5)	71.0 (64.0–78.0)
District of Columbia	82.8 (77.8–87.7)	60.6 (55.3–65.9)	11.1 (7.1–15.0)	76.6 (70.5–82.6)
Florida	76.7 (73.8–79.6)	50.8 (47.4–54.3)	7.6 (5.8–9.4)	73.0 (68.8–77.3)
Georgia	80.6 (76.8–84.3)	54.5 (50.2–58.9)	7.0 (4.7–9.2)	68.8 (63.6–74.1)
Hawaii	72.7 (65.9–79.6)	49.7 (43.1–56.4)	8.4 (3.9–13.0)	70.2 (60.7–79.6)
Idaho	76.9 (71.9–82.0)	53.1 (47.6–58.6)	6.3 (3.3–9.4)	59.9 (52.5–67.3)
Illinois	76.1 (73.0–79.3)	50.8 (46.9–54.7)	9.5 (7.4–11.7)	71.7 (67.1–76.3)
Indiana	75.7 (71.5–79.9)	54.9 (50.1–59.7)	7.3 (4.6–10.0)	76.4 (71.8–80.9)
Iowa	75.2 (70.0–80.5)	53.9 (49.5–58.2)	9.8 (6.7–12.9)	73.3 (68.5–78.2)
Kansas	77.8 (73.2–82.3)	50.7 (46.0–55.3)	7.8 (5.3–10.2)	66.8 (60.5–73.1)
Kentucky	68.9 (64.1–73.6)	48.1 (43.6–52.6)	7.1 (4.6–9.6)	73.2 (67.7–78.6)
Louisiana	76.2 (72.1–80.4)	52.8 (46.8–58.9)	4.2 (2.6–5.7)	68.8 (63.6–74.1)
Maine	80.6 (74.6–86.5)	55.5 (49.4–61.5)	8.3 (4.8–11.8)	80.2 (74.8–85.5)
Maryland	79.3 (73.4–85.2)	57.6 (49.8–65.5)	10.7 (6.5–14.9)	73.4 (65.8–81.0)
Massachusetts	84.1 (78.7–89.5)	57.5 (51.9–63.1)	8.4 (5.4–11.4)	79.6 (74.1–85.2)
Michigan	80.3 (76.3–84.3)	57.3 (53.3–61.4)	7.8 (5.5–10.1)	74.5 (70.8–78.3)
Minnesota	81.7 (77.6–85.8)	59.1 (54.1–64.2)	10.5 (7.3–13.7)	72.6 (67.7–77.4)
Mississippi	75.1 (70.9–79.2)	47.9 (42.6–53.2)	7.3 (5.2–9.3)	64.1 (56.9–71.3)
Missouri	76.4 (71.1–81.6)	54.1 (49.3–58.8)	7.2 (4.4–10.0)	69.8 (65.2–74.4)
Montana	73.7 (68.3–79.2)	49.3 (42.8–55.8)	6.8 (3.7–10.0)	66.7 (60.2–73.3)
Nebraska	79.7 (75.4–83.9)	56.9 (51.9–61.9)	7.6 (3.9–11.3)	66.5 (57.8–75.3)
Nevada	75.5 (70.5–80.6)	56.2 (50.6–61.9)	10.1 (6.1–14.1)	59.4 (52.6–66.2)
New Hampshire	81.9 (76.8–86.9)	55.0 (48.8–61.2)	7.7 (4.5–10.9)	78.6 (73.8–83.4)
New Jersey	80.9 (76.1–85.7)	52.1 (46.0–58.2)	8.9 (6.0–11.8)	72.5 (66.6–78.4)
New Mexico	75.5 (70.5–80.4)	55.6 (50.8–60.5)	9.9 (5.7–14.1)	67.2 (59.8–74.6)
New York	82.7 (79.9–85.5)	56.3 (52.5–60.0)	6.0 (4.2–7.8)	74.5 (70.5–78.6)
North Carolina	74.4 (70.8–78.0)	51.0 (46.8–55.2)	6.6 (4.7–8.6)	72.5 (68.1–77.0)
North Dakota	81.7 (77.9–85.5)	55.3 (49.6–61.0)	8.1 (5.7–10.5)	67.1 (60.9–73.2)
Ohio	78.6 (75.3–81.9)	51.7 (48.4–55.0)	5.8 (4.1–7.6)	74.2 (69.8–78.5)
Oklahoma	74.0 (69.5–78.6)	54.2 (49.8–58.6)	8.5 (6.0–11.0)	63.0 (57.0–68.9)
Oregon	80.6 (75.2–86.0)	57.9 (51.8–64.0)	8.4 (5.7–11.0)	65.8 (59.2–72.4)
Pennsylvania	73.6 (70.2–77.0)	49.5 (45.7–53.3)	6.4 (4.5–8.3)	75.1 (71.2–79.0)
Rhode Island	78.9 (73.1–84.8)	59.6 (53.2–65.9)	6.4 (3.4–9.4)	78.0 (70.7–85.3)
South Carolina	78.4 (73.7–83.2)	50.8 (46.1–55.4)	4.7 (2.6–6.8)	66.4 (60.5–72.2)
South Dakota	81.3 (76.5–86.1)	57.6 (52.9–62.3)	8.7 (5.8–11.6)	69.0 (62.5–75.5)
Tennessee	72.7 (68.5–76.8)	52.7 (48.3–57.1)	7.5 (5.1–9.9)	72.5 (67.7–77.2)
Texas	77.6 (74.9–80.4)	53.8 (50.9–56.7)	7.2 (5.4–8.9)	65.6 (61.8–69.4)
Utah	72.2 (64.9–79.5)	53.9 (44.0–63.9)	10.7 (3.5–17.9)	69.4 (61.2–77.6)
Vermont	79.7 (75.4–84.0)	57.3 (51.5–63.0)	8.6 (5.4–11.8)	78.4 (73.7–83.2)
Virginia	77.4 (72.6–82.2)	50.8 (45.8–55.8)	6.3 (3.6–8.9)	72.7 (67.8–77.7)
Washington	75.1 (69.6–80.6)	51.5 (45.3–57.6)	8.0 (4.8–11.3)	67.1 (60.2–74.1)
West Virginia	70.3 (66.0–74.6)	48.6 (43.9–53.2)	3.9 (1.9–5.9)	71.5 (65.5–77.5)
Wisconsin	76.6 (72.5–80.6)	54.3 (49.7–59.0)	7.0 (4.6–9.4)	81.7 (77.5–85.9)
Wyoming	78.4 (74.4–82.3)	52.6 (46.7–58.4)	6.9 (4.3–9.4)	66.3 (60.5–72.0)

Abbreviation: CI, confidence interval.

a Interest in quitting was defined as current smokers who reported 2–10 on a 10-point scale ranging from 1 (not at all interested) to 10 (extremely interested) among all current smokers (unweighted n = 22,163).

b Past-year quit attempts were defined as current smokers who reported that they had stopped smoking for at least 1 day or made a serious attempt to stop smoking even for <1 day within the past year and former smokers who quit within the past year among all current smokers and former smokers who quit within the past year (unweighted n = 25,850).

c Recent smoking cessation was defined as quitting smoking within the past year for ≥6 months among current smokers who smoked for ≥2 years and former smokers who quit during the past year (unweighted n = 25,507).

d Receipt of medical doctor’s advice to quit smoking was reported among current smokers who visited a medical doctor within the past year and among former smokers who visited a medical doctor within the year before quitting (unweighted n = 17,247).

**Figure:**
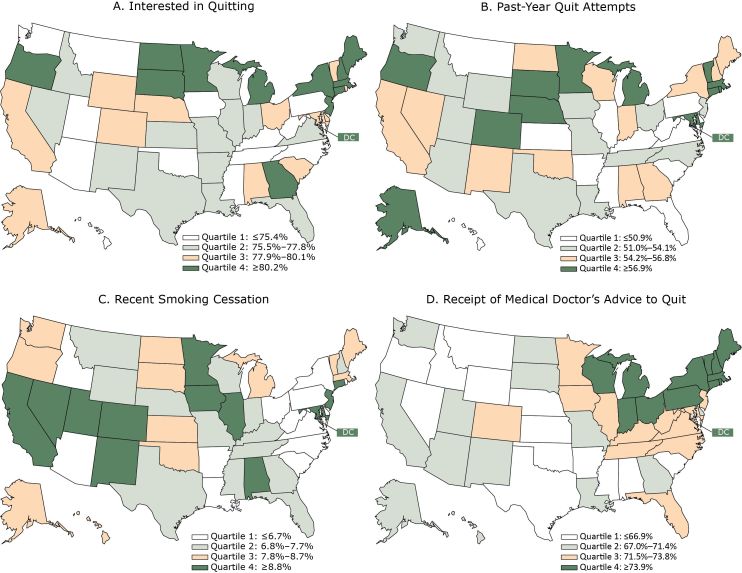
State-level prevalence of interest in quitting smoking, past-year quit attempts, recent smoking cessation, and receipt of a medical doctor’s advice to quit smoking, by quartile — Tobacco Use Supplement to the Current Population Survey, United States, 2014–2015. Panel A: Interested in quitting was defined as current smokers who reported from 2 to 10 on a 10-point scale ranging from 1 (not at all interested) to 10 (extremely interested) among all current smokers (unweighted n = 22,163). Panel B: Past-year quit attempts were defined as current smokers who reported that they had stopped smoking for at least 1 day or made a serious attempt to stop smoking even for <1 day within the past year and former smokers who quit within the past year among all current smokers and former smokers who quit within the past year (unweighted n = 25,850). Panel C: Recent smoking cessation was defined as quitting smoking within the past year for ≥6 months among current smokers who smoked for ≥2 years and former smokers who quit during the past year (unweighted n = 25,507). Panel D: Receipt of medical doctor’s advice to quit smoking was determined among current smokers who visited a medical doctor within the past year and among former smokers who visited a medical doctor within the year before quitting (unweighted n = 17,247).

**Table:** 

State	Panel A. Interested in Quitting, Quartile^a^	Panel B. Past-Year Quit Attempts, Quartile^b^	Panel C. Recent Smoking Cessation, Quartile^c^	Panel D. Receipt of Medical Doctor’s Advice to Quit, Quartile^d^
Alabama	3	3	4	1
Alaska	3	4	3	2
Arizona	1	2	1	2
Arkansas	2	1	1	1
California	3	3	4	2
Colorado	3	4	4	3
Connecticut	4	4	4	4
Delaware	3	1	1	2
District of Columbia	4	4	4	4
Florida	2	1	2	3
Georgia	4	3	2	2
Hawaii	1	1	3	2
Idaho	2	2	1	1
Illinois	2	1	4	3
Indiana	2	3	2	4
Iowa	1	2	4	3
Kansas	2	1	3	1
Kentucky	1	1	2	3
Louisiana	2	2	1	2
Maine	4	3	3	4
Maryland	3	4	4	3
Massachusetts	4	4	3	4
Michigan	4	4	3	4
Minnesota	4	4	4	3
Mississippi	1	1	2	1
Missouri	2	2	2	2
Montana	1	1	2	1
Nebraska	3	4	2	1
Nevada	2	3	4	1
New Hampshire	4	3	2	4
New Jersey	4	2	4	3
New Mexico	2	3	4	2
New York	4	3	1	4
North Carolina	1	2	1	3
North Dakota	4	3	3	2
Ohio	3	2	1	4
Oklahoma	1	3	3	1
Oregon	4	4	3	1
Pennsylvania	1	1	1	4
Rhode Island	3	4	1	4
South Carolina	3	1	1	1
South Dakota	4	4	3	2
Tennessee	1	2	2	3
Texas	2	2	2	1
Utah	1	2	4	2
Vermont	3	4	3	4
Virginia	2	1	1	3
Washington	1	2	3	2
West Virginia	1	1	1	3
Wisconsin	2	3	2	4
Wyoming	3	2	2	1

^a^ Quartile 1, ≤75.4%; quartile 2, 75.5%–77.8%; quartile 3: 77.9%–80.1%; and quartile 4: ≥80.2%.

^b^ Quartile 1, ≤50.9%; quartile 2, 51.0%–54.1%; quartile 3, 54.2%–56.8%; and quartile 4, ≥56.9%.

^c^ Quartile 1, ≤6.7%; quartile 2, 6.8%–7.7%; quartile 3, 7.8%–8.7%; and quartile 4, ≥8.8%.

^d^ Quartile 1, ≤66.9%; quartile 2, 67.0%–71.4%; quartile 3, 71.5%–73.8%; and quartile 4, ≥73.9%.

The proportion of current and former smokers who made past-year quit attempts ranged from 42.7% (35.9%–49.5%) in Delaware to 62.1% (56.7%–67.5%) in Alaska. Eight of 13 states in the lowest quartile (≤50.9%) (Figure, panel B) were in the South (Arkansas, Delaware, Florida, Kentucky, Mississippi, South Carolina, Virginia, West Virginia), and 8 of 13 in the highest quartile (≥56.9%) were in the Midwest (Michigan, Minnesota, Nebraska, South Dakota) and Northeast (Connecticut, Massachusetts, Rhode Island, Vermont).

The proportion of smokers who recently successfully quit ranged from 3.9% (1.9%–5.9%) in West Virginia to 11.1% (7.1%–15.0%) in DC. Seven of 13 states in the lowest quartile (≤6.7%) (Figure, panel C) were in the South (Arkansas, Delaware, Louisiana, North Carolina, South Carolina, Virginia, West Virginia). Five of 13 states in the highest quartile (≥8.8%) were in the West (California, Colorado, Nevada, New Mexico, Utah).

The proportion of smokers who received advice to quit from a medical doctor ranged from 59.4% (52.6%–66.2%) in Nevada to 81.7% (77.5%–85.9%) in Wisconsin. Six of 13 states in the lowest quartile (≤66.9%) (Figure, panel D) were in the South (Alabama, Arkansas, Mississippi, Oklahoma, South Carolina, Texas), and 8 of 13 in the highest quartile (≥73.9%) were in the Northeast (Connecticut, Maine, Massachusetts, New Hampshire, New York, Pennsylvania, Rhode Island, Vermont).

## Discussion

Consistent with national estimates (2), this study found that at least two-thirds of adult cigarette smokers in all states and DC expressed at least some interest in quitting. The prevalence of interest in quitting, past-year quit attempts, recent successful cessation, and receipt of quit advice from a doctor varied substantially across states during 2014–2015.

The marked variations observed across states may be attributable, in part, to differences among states in the presence of proven population-based interventions (eg, smoke-free policies), state tobacco control program funding levels, and access to proven cessation treatments such as counseling and medication (7–9). However, even jurisdictions reporting the highest prevalence of these behaviors could still benefit from improvement; in these jurisdictions, more than one-third of smokers had not made a past-year quit attempt, almost 9 in 10 smokers had not recently succeeded in quitting, and about 1 in 5 smokers who saw a medical doctor in the past year were not advised to quit. Moreover, about half of states in the lowest quartile for each measure were in the South. Together, these results reinforce the importance of ensuring equity in implementation of proven population-based interventions, particularly in states with the greatest burden of smoking and the lowest rates of cessation. These findings also underscore the importance of comprehensive state tobacco control programs and barrier-free, proactively promoted access to cessation treatments to help smokers quit successfully (10,11).

This study is subject to limitations. First, data were self-reported, and neither smoking nor cessation was validated biochemically. However, self-reported smoking status correlates with serum cotinine measurements (12). Second, the 2014–2015 TUS-CPS did not assess smokers’ use of individual methods of quitting (eg, quitlines, medications). Third, the study did not assess other factors (eg, demographics, insurance status) that could contribute to variations in the assessed measures.

Cessation behaviors among US adult cigarette smokers vary substantially by state. Opportunities exist to accelerate the implementation of proven population-based interventions and to increase smokers’ access to and use of proven cessation treatments to motivate and help more smokers to quit (1,8).
